# North-American Norms for Name Disagreement: Pictorial Stimuli Naming Discrepancies

**DOI:** 10.1371/journal.pone.0047802

**Published:** 2012-10-25

**Authors:** Mary O’Sullivan, Martin Lepage, Maria Bouras, Tina Montreuil, Mathieu B. Brodeur

**Affiliations:** 1 Department of Kinesiology, McGill University, Montréal, Québec, Canada; 2 Douglas Mental Health University Institute, & Department of Psychiatry, McGill University, Montréal, Québec, Canada; 3 Department of Psychology, Concordia University, Montréal, Québec, Canada; 4 Department of Psychology, Université du Québec à Montréal, Montréal, Québec, Canada; University of Leicester, United Kingdom

## Abstract

Pictorial stimuli are commonly used by scientists to explore central processes; including memory, attention, and language. Pictures that have been collected and put into sets for these purposes often contain visual ambiguities that lead to name disagreement amongst subjects. In the present work, we propose new norms which reflect these sources of name disagreement, and we apply this method to two sets of pictures: the Snodgrass and Vanderwart (S&V) set and the Bank of Standardized Stimuli (BOSS). Naming responses of the presented pictures were classified within response categories based on whether they were correct, incorrect, or equivocal. To characterize the naming strategy where an alternative name was being used, responses were further divided into different sub-categories that reflected various sources of name disagreement. Naming strategies were also compared across the two sets of stimuli. Results showed that the pictures of the S&V set and the BOSS were more likely to elicit alternative specific and equivocal names, respectively. It was also found that the use of incorrect names was not significantly different across stimulus sets but that errors were more likely caused by visual ambiguity in the S&V set and by a misuse of names in the BOSS. Norms for name disagreement presented in this paper are useful for subsequent research for their categorization and elucidation of name disagreement that occurs when choosing visual stimuli from one or both stimulus sets. The sources of disagreement should be examined carefully as they help to provide an explanation of errors and inconsistencies of many concepts during picture naming tasks.

## Introduction

Scientists commonly manipulate pictures and use them as stimuli to explore central processes including memory, attention, and language. However, scientists not only need pictures, they need pictures with very specific features that fit their experimental conditions. To fulfill this requirement, sets of pictures were built and normalized according to different features [1]–[7]. In the creation of their normative picture set, Snodgrass and Vanderwart [5] gathered and standardized 260 line drawings depicting various concepts - the S&V set. To ensure the pictures adequately reflected the concepts, Snodgrass and Vanderwart [5] asked their subjects to name concepts characterized by the pictures presented. A high consensus regarding a specific name, reflected by the greatest percentage of people giving this name, is considered a good indicator of quality of stimulus and “interlingua between pictures” [8]. The name with the highest percentage of agreement for each concept is labelled the “modal name” [9] and is used as the standard of comparison for the remaining names given to that picture.

The consensus reached for a picture gives only a partial indication of how a subject perceives it - much more information can be inferred by examining the other responses (e.g., [10]). For instance, the normalization procedure allows subjects to write DKO (i.e., don’t know object) when they were unable to identify the concept, DKN (i.e., don’t know name) when they did not know the name of the concept, and TOT (i.e., tip of the tongue) when the concept name did not immediately come to mind [5]. The statistics gathered from these types of responses are rarely used, but are of great importance, as they provide insight regarding task-to-task consistencies or inconsistencies in naming and imaging failures [9]; the statistics also indicate how well the picture evokes the concept. Moreover, analyses of the alternative (i.e., non-modal) and erroneous names brings additional information [11]. In their seminal paper, Snodgrass and Vanderwart [5] used the statistical *H* value as a second measure of name agreement. The *H* value is computed by taking into account the number of different names given for each picture and the proportion of subjects that gave each name. It thus provides more information than the calculated percentage name agreement regarding the *distribution* of names given to a concept across subjects. Based on the *H* value, the relative extent of the name variety given to each picture is known.

The *H* value, although used as the “primary measure” of name agreement, does not provide insight as to why several concepts were given different names, and why, when provided with the same names, a consensus was not reached. Snodgrass and Vanderwart [5] explored this issue by classifying the alternative names within what they called “sources of name disagreement.” They used four classes with which to sort these answers; these classes included: synonyms, coordinates (i.e., different exemplars of the same response category), superordinates (i.e., of greater specificity than the modal name), and subordinates (i.e., subclass of the concept pictured). Unfortunately, sources of name disagreement were only made available as a function of the concept category (e.g., furniture, food, animals) and not as a function of each concept. Moreover, it is unclear as to how incorrect names were classified, as they did not fit into any of the proposed classifications. Some concepts in the Snodgrass and Vanderwart [5] study were misnamed/misperceived; for instance, the name agreement for the asparagus was only 69%. This is mainly due to visual ambiguities and therefore confusion arose with other physically similar concepts (e.g., twig, branch, pine tree). Similar errors due to visual ambiguity were observed with a few other pictures, (doll named little girl, fox named wolf, cloud named bush, button named wheel, etc.). Accordingly, name agreement applies to the pictures and not to the concepts. Otherwise stated, the 69% of name agreement for the asparagus does not represent the consensus reached about the concept of asparagus, but rather, the consensus reached by the picture itself that was created for the S&V set. Other sources of name disagreement were proposed after Snodgrass and Vanderwart [5]. Cycowicz et al. [9] added new response categories; including component (name reflecting a part of the concept), failure (incorrect concept) and non-object (e.g., an action). Adlington et al. [1] added only the incorrect category. Vitkovitch and Tyrrell [12] categorized the S&V pictures as either correct and incorrect names. The correct names could be abbreviated (“phone” for the telephone) or elaborated (“wristwatch” for the watch).

Cases of visual ambiguity leading to a name disagreement, like the asparagus, are infrequent. This rarity may explain why the problem of naming failure has not resonated much in the scientific community except in studies with children [9] or on naming latencies [12], [13]. Naming failure has, nevertheless, become increasingly prevalent in more recent normative sets. Recent sets have sought to increase the number of pictures included in the S&V set, so that a wider variety of studies can be conducted [2], [9], [14]. However, having more pictures comes at the expense of a reduction of modal name agreement and an increase of the *H* value. It is therefore often the case that these additional pictures include concepts that are recognizable but not always easily named. For instance, Cycowicz and colleagues [9] reported a name agreement of 67.44% for an additional set of 61 pictures (set 2), and 73.18% for another additional set of 79 pictures (set 3). The name agreement for these two sets was far below the modal name agreement obtained for the 260 S&V original pictures. As another example, Bonin and colleagues [2] have created a set of 299 new pictures to complement the S&V set and they obtained a mean modal name agreement of 77.4%.

Name disagreement may also become an issue with sets composed of photo stimuli [1], [3], [6], [7]. Photo stimuli include much detail and color that, while usually helpful, could sometimes cause subjects to take into account idiosyncratic features of the concept and elicit a variety of names which differ from the modal name [3]. For instance, the box in the Bank Of Standardized Stimuli (BOSS) [3] has a decorative design, and was consequently named “gift box” and “decorative box” by 26% and 10% of the subjects, respectively. Since no such details were found in the line-drawn box of the S&V set, subjects were inclined to simply name it “box”. Moreover, the evolution of technology and market products could reflect the lower modal name agreement, as products have become more diversified and the need for specificity and detail when naming a concept has become commonplace (e.g., naming a PDA “Blackberry”).

The need for classification of norms is imperative in order to reflect reasons for name disagreement. These norms provide better characterization of the stimuli and indicate how accurately they are recognized. For example, for a concept with a low modal name agreement, these norms offer insight as to whether the concept is often confused with other similar concepts, or, rather, the low name agreement is due to various synonyms existing for the concept’s name. Computing the sources of name disagreement is also critical because of its consistent influence on naming latency [8], [13], [15]–[17]. All steps of the naming process described by Humphreys et al. [18] are subject to such influence. For instance, disagreement due to visual ambiguity of the picture will likely influence the analysis of the concept’s appearance and the recovering of stored structural knowledge. On the other hand, the amount of information that is extracted from the picture will instead influence the step of activation of semantic information. Finally, the number of other names activated by the picture will affect the ease of retrieving the correct name. Vitkovitch and Tyrrell [12] showed that the influence of disagreement on the different steps depends on its underlying cause (i.e., source). Results from their two experiments indicated that disagreement due to the use of incorrect names slowed down the naming process because of difficulties encountered at or before a structural analysis stage of recognition. In contrast, disagreement due to the use of correct alternative names affected the naming process at the name retrieval stage, thus later in the process. Sources of name disagreement thus complement name agreement, one of the strongest predictor of naming latency [16], by tracking the processes and the representations that are involved in concept naming.

In the present work, we propose new norms which reflect sources of name disagreement, and we apply this method to two sets of pictures: the S&V set [5] and the BOSS [3]. The alternative names in both sets were first categorized as correct, incorrect, or equivocal. A correct alternative name could be the word “stick” given to a picture with “branch” as the modal name. An incorrect name would rather be the word “bagel” given to the picture of a donut. As for the equivocal name, it could be words such as “hold presser” or “druidck” which were likely made-up by the subject and for which correctness could unlikely be determined. In a second step, the names were placed within another response category as a function of the naming strategy the subjects likely used to provide that name. Subsequently, the norms were compared across the S&V set and the BOSS.

## Methods

### Sets of Stimuli

The first set included in the study, the S&V set, consists of 260 line drawings of various concrete concepts, such as objects, animals, body parts, vehicles, etc. [5]. The drawings are black outlines on a white background; they were selected to provide exemplars from widely studied semantic categories. The concepts in S&V set were gathered and line-drawn based on a set of established guidelines. The criteria included: the drawing having realistic details, being the most typical representation of the concept, and being consistent with the complexity of the real-life concept. Each concept was further subject to criteria for orientation; for example, animals are shown sideways, and concepts that have varying up-down orientation (e.g. fork) are drawn with the functional end down.

The second set, the BOSS, is a large set of 480 high quality color photo stimuli of common (to North America) concepts [3] (https://sites.google.com/site/bosstimuli/). Photo-stimuli were created through a 5-step procedure. The procedure went as follows: (1) common concepts were gathered and photographed; (2) concept cut-out (from original photo); (3) picture editing (removing stains, brand names, logos, etc.); (4) luminance adjustments (adjusting color, lightness, and contrast in a way that improved the concept visibility); and (5) resizing (resize concept to standard size and place in a frame of 2000×2000 pixels). From the original BOSS, 13 concepts were excluded in our study due to imprecision in naming of the concept that could be considered as incorrect (e.g. pet carrier named “animal cage”). The 13 excluded picture files were: babybottle, babyseat, bubblesblower, blender, gluetube, jar02, muffintin02, animalcage, soil, tractor01b, wallet04, wirecutter02, wirestripper.

Norms of the S&V set were collected from 42 subjects, all of whom were volunteers from introductory Psychology courses. There was approximately equal numbers of male and female participants. Norms for the BOSS set were collected from 39 subjects (22 females) aged, on average, 33.6 (±12.7) years old. The number of subjects included in normative studies is highly variable and ranges from approximately 20 [2], [15], [19], [20] to over 100 [4], [10], [21], [22]. Groups of 30 to 50 subjects nevertheless represent the sample size that has been the most widely used thus far in normative studies [3], [6], [9], [14], [23]–[28].

### Procedure

In the two studies, random sequences of stimuli were projected on a screen and presented to subgroups of subjects for the normalization procedure. Each subject was given a data response sheet with the appropriate amount of lines for recording names of the concepts presented. Instructions for both studies were given orally to the subjects and they were written on a separate sheet in the BOSS study. When naming the concept, subjects were instructed to: “Identify the concept as briefly and as unambiguously as possible by writing only one name, the first name that comes to mind.” Moreover, subjects in both studies were instructed to write DKO (don’t know object) if they did not know the object. If they knew the object but did not know its name, they were instructed to write DKN (don’t know name), and, if they were unable to momentarily retrieve the name of the object but knew it, to write TOT (tip-of-the-tongue).

The present study proposes new statistics computed on the alternative, non-modal names to elucidate the reasons that may account for labelling concepts in a manner that differed from the modal name. Each alternative name was placed within one of three predefined response categories: equivocal, correct, or incorrect. Names classified into each of these categories were further classified into specific sub-categories for name disagreement.

Response categorization was performed by two native English-speaking evaluators (the first and third authors) who applied the above-described rules to reach a consensus regarding the source of name disagreement for each response. Discrepancies between the two evaluators were mediated by a third critic and a consensus was reached.

### Measures of Agreement

#### Modal name agreement

Naming frequency for each name provided for each concept, as well as the DKO, DKN, and TOT responses, were calculated and converted to percentages. The name reaching the highest percentage was the modal name, and its percentage reflected the modal name agreement. These statistical calculations have already been undertaken in the S&V and the BOSS studies. In the BOSS study, it happened on rare occasions that two names given to one concept had equivalent name agreement percentage, such as “vice grip” and “wrench”, or “USB key” and “USB”. In such a case, the most precise name, “vice grip”, and “USB key” respectively, were designated as the modal name. Furthermore, in the BOSS study, answers for names were ruled as “different” from the modal name in all responses composed of dissimilar words with the exception of two cases. First, the adjective that described a state (e.g., empty glass) or a feature that was totally irrelevant for the identity of the concept (e.g., white candle) was left out. The adjective counted as long as it provided relevant information. For instance, the adjective “girl” in the response “girl sock” is highly relevant, since it defines a specific type of sock. The adjective can also be subjective; for example, “fancy” in the response “fancy glass” may refer to a type of glass for the subject. In fact, only 7 adjectives were discarded over the 20,760 responses from the 538 stimuli originally composing the BOSS. The second difference between names that were not taken into account was the word order in composite names. Thus, “bottle of beer” and “beer bottle” were counted as the same name. The system of modal name identification in the BOSS contrasts that of the S&V study. Snodgrass and Vanderwart [5] computed the model name by calculating the number of modal names over the number of input including DKO, DKN, TOT; whereas modal names in the BOSS excluded the DKO, DKN, TOT inputs. In the present study, modal name agreement was computed by excluding the DKO, DKN, and TOT responses.

#### Modal noun and adjective/modifying noun agreement

The modal name agreement does not systematically or necessarily reflect the real use of the word. For example, “chair” is the modal name for the picture of a chair in the BOSS - with 51% of agreement only. However, the word “chair” was used in all alternative names (e.g., “desk chair”, “office chair”, “rolly chair”, “swivel chair”, and “computer chair”), leading to an agreement for the word “chair” of 100%. The agreement for each modal noun was thus computed independently from the adjective or other words accompanying the noun. The agreement of the adjective and modifying nouns (e.g., the word “beer” in the name “beer bottle”) of composite names was also computed. The noun and adjective/modifying noun agreement can be useful as it avoids name disagreements due to specificity.

#### Statistic H

Another norm, the statistic *H*, or *H* value, was computed in addition to the name agreement. The *H* value is sensitive to the number and weight of alternative names. It was computed with the following formula:
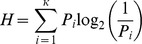
Where *k* refers to the number of different names given to each picture and excludes the DKN, DKO, and TOT responses. *P_i_* is the proportion of subjects who gave a name for each concept. As a result of the exclusion of the DKN, DKO, and TOT responses, it must be noted that this proportion varies across the concepts. The *H* value of a concept with a unique name and no alternatives is 0. The *H* value of a concept with only two names provided with an equivalent frequency is 1.00. This value is smaller for an alternative that is provided at a lower frequency rate. On the other hand, the *H* value increases as a function of the number of alternatives. For instance, one modal name of 50% frequency and two alternatives of 25% frequency each give an *H* value of 1.50. The *H* value is usually highly correlated with the modal name agreement. Their correlations were of −.907 and −.960 in the S&V set [9] and the BOSS [3], respectively. Other studies using the S&V set also reported high correlations (−.916 [2], −.967 [29], −.952 [30], −.830 [8]).

### Measures of Disagreement

All names that differed from the modal names were alternative names or non-modal names. Names were each assessed through a two-step procedure to try and determine why they were used instead of the modal name.

#### Equivocal names

The first step of the response categorization process was to determine if the name is an English word that consistently refers to the same concept or type of concept. If so, the name was submitted to step 2. If not, the name was considered as equivocal and placed within one of the following three sub-categories: unknown, idiosyncratic, or non-object.

Unknown names were words that cannot be found in the English dictionary. Their meanings were impossible to decipher definitively (e.g. “steering wheel zoso” for a TV antenna). Unknown names also included words from another language such as “stylo” for the fountain pen.

Names classified as idiosyncratic were not found in the English dictionary but likely reflected personal wording that was not of a proper or common use, such as “muffin form”, “wine opener”, or “serving fork”. These names generated only a few pictures when searched using Google Images or, they generated many but inconsistent pictures (e.g., “cookie maker” for the cookie cutter was linked to different kind of objects). The idiosyncratic names were generally compound names that had an understandable meaning associated with a specific and consistent concept. For example, the paint can was named “jar of paint”. This was an understandable term, but since this word is improper, it cannot be classified in the sub-categories of step 2.

Some of the alternative names referred to an action (e.g., “fishing”), a person (e.g., “daddy”), an adjective (e.g., “musical”), or an intangible concept (e.g., “badminton”). These names could not be classified within the response categories of step 2.

#### Correct names

The second step involved determining whether the name was correct or incorrect. Correct names were words that reflected appropriately the concepts presented. They were classified within one of the following four sub-categories: Unspecific, specific, mixed, or synonym. The classification of the name as correct or incorrect was always made relative to the real identity of the concept depicted in the photo. The sub-classification was made relative to the modal name.

This response category applied to names that left out distinctive information found in the modal name. This occurred when a subject named a concept by the general semantic category or broader concept under which it fell. The unspecific names were words like “tool” to identify a chisel, or “fruit” to identify a blackberry. They could also be more precise than a semantic category and refer to a general type of concept. For instance, subjects identified the “wine glass” as a glass. Finally, a name was classified as unspecific when one word of a compound modal name was missing, even when this missing word did not change the meaning of the name, such as “cell” for “cell phone”. The remaining word must have, however, remained the same. Therefore, “magnifier” for the modal name “magnifying glass” was not classified as unspecific but as synonym.

This classification was for names that provided more information than the modal name and, as such, it was opposed to unspecific names. In many cases, the specification was achieved with the addition of words to the modal name, even when these words were of no real relevance. Therefore, the name “ball of yarn” for the yarn and “potato peeler” for the peeler were classified as specific names. The specific names were also entirely different from the modal names as long as they provided more information regarding the concept, such as “brie” instead of “cheese”, however, the specification had to be true. For instance, a few subjects specified the brand mark of the concept, such as “pamper” for the diaper; this was considered a specification so long as it was true. The same rules applied to names referring to the specific content of a concept that could not be seen. This was the case with the bottle or tube named “bleach bottle” or “glue tube”, respectively. Specifications sometimes led to incorrect names when it changed the nature of the concept, such as “feather pen” for the pen. In such cases, the alternative name was more specific but wrong, thus classified within the “incorrect” response category.

Often, the elicited names missed information while it also added new information about the concept. The “two of spades” name for the playing card is an example of this mixture of the unspecific/specific response category. The specific information was sometimes inherent to the name but there were a few cases in which the mixed response category could be considered. First, it was occasionally the case where alternative names referred to a part/function of the concept that is different from the part/function identified by the modal name. Names such as “ice scraper” for the snowbrush, or “trimmer” for the electric razor were classified under the mixed response category. For the case of “dried fruit” for the raisin, “dried fruit" should *not* be classified as a mixed despite the “dried” for the specification and the “fruit” for the unspecific information; this is because raisins are inherently dried and therefore, “dried fruit” was classified as unspecific.

This classification of name disagreement applied to names that were different from the modal name but which referred to the same concept. The synonyms provided no more or less information than the modal name, such as, “watering jug” and “watering can”, or “mobile phone” and “cell phone”. “Nail clipper” and “nail cutter” were synonyms but “nail scissors” was another concept and was therefore incorrect. Furthermore, “candlestick” was a synonym of “candle” and not a specific because “candle” and “stick” form a single word. Synonyms could also have more or less words than the modal name; however, additional words must not had provided more information and no word must have been the same across the two names. Therefore, “laptop” was a synonym for “portable computer” but “portable laptop” was considered as a specific name.

#### Incorrect names

Names were incorrect when they referred to a different concept than the one depicted in the picture. Incorrect names were classified within two sub-categories consisting of ‘physically similar’ or ‘physically dissimilar’ concepts. Ideally, this step required information on the real identity of the concept, its brand mark, and its composite materials.

Some incorrect names referred to concepts that were physically identical to the modal concept. The subject must therefore have chosen amongst several possibilities. For instance, the plastic glass was identified as a “garbage bin”, and the nasal spray as a “dropper bottle”. These examples are cases of extreme ambiguity because no information contradicted the alternative name. Similarity was also considered for concepts that resembled the object but were not totally identical. In such case, similarity was arbitrarily determined and had to be decided independently from the semantic common point between the concepts evoked by the modal and alternative names. Therefore, “riding helmet” (used for equestrian sport) for a bike helmet was not considered as physically similar. A name was also categorized as incorrect when it presented false information about the concept and the material composing it, even when this concept was correctly identified. This was the case of the medal named “olympic medal”, or “crystal wine glass” for a simple wine glass. Finally, when a subject correctly named the concept but assigned it to an incorrect brand mark (e.g., “speedstick” for a deodorant from another brand mark), the name was incorrect and physically similar. The only exceptions were brand marks that are assimilated to the concept identity in the community, such as “Kleenex” for tissue box.

Incorrect names that were classified as physically different were words referring to concepts bearing no resemblance to the concepts depicted in the picture. It was sometimes the case that the physically different incorrect name was a coordinate, [5], [9] semantically related to the concept such as “lego” for puzzle piece; however, the modal name and the alternative name must not have been visually ambiguous to be categorized as incorrect and physically different. The physically different sub-category was thus achieved independently from the semantic relationship between concepts.

### Analyses

Analyses were done to (1) characterize each picture of the two sets (BOSS and S&V), (2) to see how the alternative names are distributed across the response categories (equivocal, correct, incorrect) and sub-categories (idiosyncratic, unspecific, mixed, etc.), and (3) to see if these distributions differ across sets.

Analyses were conducted on concepts, not subjects. The percentage of subjects giving each name reflects the concept naming “performance”. Modal name agreements and *H* values were compared between sets by two independent sample *t*-tests. The interaction for testing the differences of response categories between sets was examined using an ANOVA - the set was the between-“concept” factor, and the response category was the within-“concept” factor. In case of interaction, the same ANOVA was run for each response category with the sub-category as the within-“concept” factor and independent sample *t*-tests were completed for each response category.

## Results

All norms computed from the names given by subjects are presented in Table 1. Norms in this Table reflect the mean percentage for all concepts of each set. The norms for each concept can be viewed in Supporting Information S1 and S2.

**Table 1:** Norms for the S&V set and the BOSS.

	S&V set		BOSS	
Variables	Mean	SD	Mean	SD
Modal name agreement	88.0%	13.7%	65.0%	23.0%
Modal noun agreement	89.6%	12.7%	77.4%	19.4%
Modal adjective/modifying noun agreement*	72.5%	15.4%	68.4%	19.8%
*H* value	0.56	0.53	1.61	0.98
DKO	0.3%	1.2%	1.6%	3.4%
DKN	0.7%	2.1%	5.7%	7.6%
TOT	0.7%	1.7%	1.6%	2.6%
Equivocal	2.2%	9.4%	10.7%	17.8%
- Unknown	0.5%	3.3%	3.7%	11.2%
- Idiosyncratic	1.6%	8.7%	5.6%	12.5%
- Non-object	0.0%	0.0%	1.4%	6.8%
Correct	68.2%	40.2%	57.3%	31.7%
- Specific	38.9%	43.2%	25.5%	31.5%
- Unspecific	19.9%	34.6%	21.1%	26.7%
- Mixed	3.4%	13.4%	4.1%	10.5%
- Synonym	6.1%	20.1%	6.7%	15.9%
Incorrect	29.6%	39.4%	32.0%	29.4%
- Physically similar	18.3%	33.1%	12.8%	21.1%
- Physically different	11.3%	25.3%	19.2%	23.8%

DKO = Don’t know object; DKN = Don’t know name; TOT = Tip-of-the-tongue.

* Includes only names with compound words (n = 132 in the BOSS and n = 21 in the S&V).

An independent sample *t*-test comparison showed that the modal name agreement and the *H* value were significantly lower (*t*(14.77), *p*<.0001) and higher (*t*(−16.00), *p*<.0001) for the BOSS than the S&V set, respectively. The difference between the sets was smaller for the modal noun but still significant (*t*(725) = 9.16, *p*<0.0001). Statistics on adjective/modifying noun agreement only included names that were compound names, and the difference did not reach significance. These analyses included 132 stimuli in the BOSS and 21 stimuli in the S&V set. Analysis showed that the proportion (28%) of compound names was significantly greater in the BOSS than in the S&V set (8%) (χ2 (1) = 41.0, *p*<.0001). The name agreements of the compound names were also computed for each set and resulted in 70.5% (SD: 14.8%) for the S&V set and 55.0% (SD: 20.3%) for the BOSS. These agreements were significantly different (*t*(3.36), *p* = .0010). Finally, the DKO (*t*(6.29), *p*<.0001), DKN (*t*(10.40), *p*<.0001), and TOT (*t*(−4.8), *p*<.0001) responses were all significantly more frequent in the BOSS than in the S&V set.

The first analysis conducted on the name disagreement was an ANOVA with the response category as the within-concept variable, and the set as the between-concept variable. The interaction achieved significance (*F*(2,1284) = 10.92, *p*<.0001), as well as the main effect of response category (*F*(2,1284) = 353.60, *p*<.0001). The interaction of the sub-category and the set was then statistically tested within each response category using other ANOVAs. The interaction for the equivocal category was almost significant ( *F*(2,1284) = 2.86, *p* = .0583) but the main effect of sub-category (*F*(2,1284) = 14.05, *p*<.0001) and set (*F*(1,642) = 41.43, *p*<.0001) were highly significant. The names of BOSS’ pictures were thus more frequently classified as unknown, idiosyncratic, and non-object than the names in the S&V set.

The ANOVA conducted on the correct names led to a significant interaction between sets and response category (*F*(3,1926) = 129.14, *p*<.0001), and significant main effect for each of these variables (response category: *F*(3,1926) = 9.35, *p*<.0001; set: *F*(1,642) = 13.90, *p* = .0002). The between-set differences were examined for each response category using independent sample *t*-tests. Results showed that only the use of specific names was significantly different between sets (*t*(642) = 4.45, *p*<.0001). Between-set differences for the unspecific, mixed, and synonym categories had *t*-values below.68 and were all not significant.

The interaction between sets and response category of incorrect names was significant (*F*(1,642) = 18.04, *p*<.0001) but not the main effect of response category (F(1,642) = .04, p = .8381) and set (*F*(1,642) = .75, *p* = .3863). The independent sample *t*-tests conducted on each response category showed that concepts were more likely confounded with a physically similar concept in the S&V set than in the BOSS (*t*(642) = 2.55, *p* = .0109). Conversely, concepts in the BOSS were more frequently given the name of a very different concept (physically dissimilar) (*t*(642) = −3.86, *p* = .0001).

## Discussion

In the literature, modal name agreement is used primarily for validating the use of the pictures by assuming that high name agreement reflects the correct recognition of the pictures. Giving alternative (non-modal) names to a picture, however, does not imply that the picture was not properly recognized; it depends on whether these names refer to other concepts. The objective of this study was to create and classify new norms based on the classification of the alternative names of the BOSS and S&V pictures as correct, incorrect, or equivocal. To characterize the naming strategy accounting for the use of an alternative name, non-modal names were further divided into different sub-categories reflecting various sources of name disagreement. The norms gathered in this paper will prove helpful for subsequent research when choosing visual stimuli from one or both stimulus sets, as they offer better characterization of the pictures and a greater understanding of the effects of visual stimuli on naming, categorization and recognition.

Name agreement has proven to be higher in the S&V set with an agreement of 88.0% than for the BOSS with an agreement of 65.0%. Brodeur et al. [3] argued that differences of concept selection between the BOSS and the S&V set were largely responsible for this difference. The BOSS includes only common objects whereas the S&V set also includes animals, vehicles, and body parts. The possibility of gathering concepts with higher name agreement was greater for the S&V set as it covers a wider range of potential concepts. This possibility is reduced as the number of stimuli increases. The higher number of stimuli in the BOSS and its limitation to common objects could thus account for its lower name agreement. A last variable that might have lowered the name agreement in the BOSS is the inclusion of subjects that were, on average, older than those in the Snodgrass and Vanderwart study (1980). Sirois et al. [20] have shown that name agreement decreased with age, a result further supported by Yoon et al. [10] who reported significant differences of name agreement between older and younger American adults for a significant proportion of pictures.

The present results also suggest that the higher number of compound names in the BOSS may account for the difference of name agreement between the sets. The BOSS includes 132 compound names in contrast to 21 in the S&V set. Compound names have been shown to substantially lower name agreement. For instance, Janssen et al. [31] normalized 150 line drawn pictures with modal compound names and obtained a mean name agreement of 67.5%, thus clearly below the name agreement of the S&V set which includes only a limited number of concepts with compound modal names. The name agreement of the compound names of the S&V set and the BOSS were respectively 70.5% and 55.0%, and thus clearly lower than the modal name agreement of the modal names including a single word. Norms computed for the noun agreement also supported a probable influence from the compound names on name agreement. When only considering the noun to compute the agreement, the between-set difference is cut in half, from 23.0% to 12.2% (see Table 1).

The quality of the stimuli relies to a large extent on the correctness of the alternative names. This classification could, however, only be achieved on names referring to known concepts. Actions, abstract concepts (with no physical entity), and personal wording for which the exact meaning could not be verified, had to be excluded from the correct/incorrect categorization. These equivocal names distributed across the idiosyncratic, unknown, and non-object sub-categories, deserve attention because they can indicate a specific naming strategy. Personal words were not incorrect, but rather, their exact meanings left room for ambiguity and misinterpretation; in both sets the idiosyncratic words were used more often than unknown and non-object names. However, this pattern of concept naming was definitely more frequent in the BOSS set (10.7%) than in the S&V set (2.2%). As explained above, the BOSS set includes concepts of daily use and people thus tend to use their own words, personally-used words, or combinations of words to name the concepts. This name may vary depending on the way people use the concept or on the context in which they use it. The use of personal words was also likely due to the type of concepts presented in the BOSS or the detail in the picture as compared to the drawings. This personal use of words also accounted for the lower name agreement in the BOSS and is consistent with a difficulty in finding concept names in the BOSS, as reflected by greater number of DKO, DKN, and TOT responses. With the use of equivocal names, naming difficulty is also associated with the choice of visual stimuli in the BOSS involving actions, specific details, abstract concepts, and compound names.

Of the alternative names, 68.2% and 57.3% were correct in the S&V set and the BOSS, respectively. The use of correct names in the BOSS was reduced at the expense of the use of idiosyncratic and equivocal names. Comparison of the sub-categories of correct names showed that in both sets, name diversity was due to the level of specificity in the words the subjects chose to use. The level of specificity was determined relative to the modal name; this explains why there were more specific correct names used in the S&V set than in the BOSS. This was to be expected, given that the pictures in the S&V set are more prototypal and have less detail, and therefore promote modal names that are unspecific, thus increasing the chance of giving more specific alternative names. This was the opposite case of the BOSS set where the pictorial representations of the concepts have more detail and inherently receive more specific modal names.

Names were classified as incorrect when they inadequately identified the concept. This norm is probably the most relevant one as it indicates the extent to which the depicted concepts tend to be misperceived or confused with other concepts. Our results showed that the percentage of incorrect names in the sets was low and there was no significant difference between sets, with 29.6% and 32.0% of incorrect names for the alternative names of the S&V set and the BOSS, respectively. More interestingly, it was found that concepts were more likely confounded with a physically similar concept in the S&V set than in the BOSS. The lack of details and features, such as color, in the S&V concepts have largely contributed to this confusion by leaving more room for naming a similar but incorrect concept (ex. leopard confused for tiger or jaguar). Color is known to be one of the most efficient features for discriminating stimuli in various tasks. Its impact on name agreement has also been reported by Rossion and Pourtois [19] after they compared the norms of the original S&V set and a colored version of this set. Their results showed that the *H* value of the colored set was significantly smaller, meaning that subjects used less alternative names. In contrast to the S&V concepts, BOSS concepts were more frequently given the name of a very different concept (physically dissimilar). Many of the incorrect classifications of words were due to improper use of vocabulary on the part of the subject; this biased the naming strategy toward the use of names reflecting dissimilar concepts. We could understand what the subject meant when naming the concept; however it was incorrect and instead applied to a different concept (e.g., “paper cutter” for a hole punch). The improper use of words was also likely responsible for the greater use of idiosyncratic names.

In future research, scientists requiring visual stimuli could rely on the S&V set, the BOSS, or any other sets of pictures, although they should make their final selection by considering not only their specific research needs but also the strengths and weaknesses of the different sets. Weaknesses in the S&V set are mainly due to the “modality” (illustration, line-drawing) causing increased visual ambiguity and confusion with resembling concepts. Ideally, visual stimuli should be unambiguous [32] but the lack of detail in the line-drawing pictures is the cost associated with using typical, pictorial representations of a concept. Simplification of a picture representation has been shown to increase the naming latency [33]. Naming latency is also known to be delayed with black-and-white line drawing compared to colored drawings [19] or colored photos [33], [34], because of a greater visual ambiguity (but see [35]). On the other hand, the S&V pictures suits well the need for experiments in which the modality of the illustration is not important and where the pictures are used as a medium for evoking a concept. In such experiments, it might be preferred to minimize details that otherwise would distract the subject and hence, bias the performance. Scientists wishing to minimize visual ambiguity should refer to the colored version of the S&V set developed by Rossion and Pourtois [19]. Like all sets of photos (see [1], [6]), the BOSS is visually less ambiguous and ecologically more valid than the S&V set; however, it is subject to the common use of personal nomenclature from subjects. This strategy necessarily causes an increased use of alternative names and lowers the modal name agreement. This weakness is partially due to the colored photo “modality” and, to a greater part, to the nature of the concepts included in the BOSS, which is limited to common concepts. More important is the high number of concepts included in the BOSS that comes at the expense of the inclusion of concepts that were necessarily more difficult to name accurately. The problem of lower modal name agreement can nonetheless easily be minimized by limiting the selection of pictures to those with the highest modal name agreement.

Norms for sources of name disagreement presented in this paper are useful for subsequent research for their categorization and elucidation of name disagreement when choosing visual stimuli from one or both stimulus sets. The sources of disagreement and misidentification should be examined carefully as they help to account for errors and inconsistencies obtained for many concepts. The sources of disagreement and misidentification also represent useful tools to better define the profile of each concept and to select concepts for particular studies accordingly. They allow the investigator to have greater number of options with respect to investigating a specific research topic. They elucidate where and how subjects will perceive objects from these sets and how one can achieve or avoid utilizing visual stimuli that are generally correct, incorrect, or equivocal.

## Supporting Information

Supporting Information S1 Sources of name disagreement of the S&V pictures.
(XLSX)

Supporting Information S2 Sources of name disagreement of the BOSS photos.
(XLSX)
